# Environmental Hazards and Behavior Change: User Perspectives on the Usability and Effectiveness of the AirRater Smartphone App

**DOI:** 10.3390/ijerph18073591

**Published:** 2021-03-30

**Authors:** Annabelle Workman, Penelope J. Jones, Amanda J. Wheeler, Sharon L. Campbell, Grant J. Williamson, Chris Lucani, David M.J.S. Bowman, Nick Cooling, Fay H. Johnston

**Affiliations:** 1 Menzies Institute for Medical Research, University of Tasmania, Hobart 7000, Australia; belle.workman@utas.edu.au (A.W.); penelope.jones@utas.edu.au (P.J.J.); Amanda.Wheeler@acu.edu.au (A.J.W.); sharon.campbell@utas.edu.au (S.L.C.); 2 Mary McKillop Institute for Health Research, Australian Catholic University, Melbourne 3065, Australia; 3 Public Health Services, Department of Health, Hobart 7000, Australia; 4 School of Natural Sciences, University of Tasmania, Hobart 7000, Australia; grant.williamson@utas.edu.au (G.J.W.); christopher.lucani@utas.edu.au (C.L.); david.bowman@utas.edu.au (D.M.J.S.B.); 5 School of Medicine, University of Tasmania, Hobart 7000, Australia; nick.cooling@utas.edu.au

**Keywords:** air pollution, pollen, smartphone app, health, behavior change

## Abstract

AirRater is a free smartphone app developed in 2015, supporting individuals to protect their health from environmental hazards. It does this by providing (i) location-specific and near real-time air quality, pollen and temperature information and (ii) personal symptom tracking functionality. This research sought to evaluate user perceptions of AirRater’s usability and effectiveness. We collected demographic data and completed semi-structured interviews with 42 AirRater users, identified emergent themes, and used two frameworks designed to understand and support behavior change—the Behavior Change Wheel (BCW) and the Protective Action Decision Model (PADM)—to interpret results. Of the 42 participants, almost half indicated that experiencing symptoms acted as a prompt for app use. Information provided by the app supported a majority of the 42 participants to make decisions and implement behaviors to protect their health irrespective of their location or context. The majority of participants also indicated that they shared information provided by the app with family, friends and/or colleagues. The evaluation also identified opportunities to improve the app. Several study limitations were identified, which impacts the generalizability of results beyond the populations studied. Despite these limitations, findings facilitated new insights into motivations for behavior change, and contribute to the existing literature investigating the potential for smartphone apps to support health protection from environmental hazards in a changing climate.

## 1. Introduction

Air pollution, aeroallergens and heat represent important environmental health hazards that can greatly impact individual and community health. With respect to air pollution, it is estimated that exposure to ambient (outdoor) air pollution was responsible for approximately 4.2 million deaths globally in 2015 alone [1]. Exposure to particulate matter (PM), a major component of air pollution, has been linked to various adverse health outcomes, including the development and worsening of cardiovascular and respiratory conditions [2,3]. These chronic health conditions that include heart failure and chronic obstructive pulmonary disease are leading causes of morbidity and mortality in developed and developing countries [4].

Aeroallergens (primarily pollen and fungal spores) are also common exposures worldwide and can provoke immune responses causing symptoms of allergic rhinitis, conjunctivitis or asthma, which are common chronic conditions across a broad range of age groups [5,6,7]. Extreme weather-related events, such as heat and cold waves, represent another important environmental exposure [8,9], with health implications that can lead to illness or death in vulnerable populations such as the elderly and children, those with pre-existing respiratory and cardiovascular conditions, as well as outdoor workers [10]. Individually and collectively, these three environmental hazards affect entire populations and can lead to large public health impacts as many people are in groups at higher risk of experiencing adverse health outcomes.

Climate change is likely to increase all of these exposures for much of the global population with flow-on effects for human health [11,12]. Climate-related projections assert an increasing risk on a large scale of more frequent and severe air pollution episodes, such as bushfires [13]. This may have substantial economic costs: health-related costs associated with smoke during the 2019–2020 bushfire season in Australia were estimated at AUD$1.95 billion (USD$1.5 billion) [14]. A changing climate will also influence pollen loads and the length of pollen seasons, increasing pollen-associated risks and conditions [15]. Additionally, climate change is projected to increase vulnerability to heat-related health risks, which already pose a high health burden in some countries [16,17,18]. In the context of these projected increases in health risks from environmental hazards in a changing climate, it is imperative to provide evidence-based interventions that support public health.

The validity of mobile health technology to support individual and community health outcomes from environmental hazards warrants further attention. The development of smartphone and mobile health (mHealth) apps to support general health, fitness and specific medical conditions has grown significantly in recent years [19]. These include a number of mHealth apps designed to provide timely and accessible data on environmental hazards, such as air quality, to support health decision-making [20,21,22]. Studies investigating individual adherence to information and/or alerts on environmental hazards report mixed results, however many consistently conclude that individuals with pre-existing health conditions are more likely to use such information to inform health decision-making [23,24,25,26,27].

Evidence from smartphone apps designed to inform health decisions in the face of environmental hazards, such as poor air quality, support this finding. For example, Licskai et al. (2013) reported improvements in symptom criteria for uncontrolled asthma through the provision of access to current and forecast air quality health index information to support asthma self-management [21]. More recently, Che et al. (2020) developed the Personalized Real-Time Air Quality Informatics System for Exposure—Hong Kong (PRAISE-HK), to provide air quality health index information to assist individuals to manage their exposure to air pollution [20]. While results on the validity of PRAISE-HK are forthcoming, its development clearly demonstrates a research interest in exploring apps as an alternative, and potentially more effective, information source and communication avenue for air quality-related information. Integrating data on additional environmental hazards, such as pollen and temperature, may enhance the utility and applicability of an mHealth app, appealing to a broader audience with more diverse pre-existing health conditions. The app, AirRater, was developed to address this gap.

### 1.1. An Introduction to AirRater

AirRater is a free smartphone app developed in 2015 that was designed to support individual health by empowering users to make more informed decisions about managing their exposures to environmental health hazards. The app gathers environmental data from government agencies and integrates the information to present users with local, near real-time data on air quality (specifically PM concentrations), total pollen count (in Tasmania and the Australian Capital Territory (ACT) only), selected pollen and fungal spore species, and air temperature (both actual and apparent). A color-coded labelled rating system assists users to interpret the air quality, pollen and temperature measurements displayed. For example, a green “Good” rating indicates a PM_2.5_ range of 0–9 micrograms per cubic meter of air (see Figures S1 and S2 in the Supplementary Material for supporting graphics and a schematic representation of the app).

Users also have the capacity to:

- identify and capture their symptoms, medication use and potential triggers associated with environmental exposures through a symptom reporting feature. The app summarizes and graphically presents key symptoms, medication use and suspected triggers over time, in conjunction with detailed environmental hazard data for a specified time period. This supports users to gain insight into personal triggers, such as a particular pollen species. The app further allows users to create personal notifications for specific environmental hazards relevant to their needs.
- view environmental data at multiple locations. The app includes a location search and pin drop function so that users can quickly and easily determine environmental hazard data in an alternative location.
- overlay numerous data maps over geographical areas. The interactive map function includes the provision of data on current bushfire activity, monitoring stations, and symptom hotspots.

More detailed presentations of AirRater’s rationale and functionality, as well as initial evaluation outcomes, are available elsewhere [28,29,30]. The app was developed by a multidisciplinary collaboration of researchers and government agencies. The original team comprised representatives from the University of Tasmania, the Environment Protection Authority (EPA) Tasmania, the Commonwealth Scientific and Industrial Research Organization (CSIRO) and the Australian National University (ANU). While the app was initially developed for Tasmania, it has progressively been introduced into other Australian jurisdictions and is now available nationally. Numerous relevant government agency and stakeholder representatives, such as state health departments, state environment departments and Asthma Australia, have been, or have increasingly become, involved in supporting the app as funders or collaborators. For example, several health departments including the Tasmanian Department of Health, the ACT Department of Health, and the Northern Territory (NT) Department of Health assist in funding the app and include references to the app on their websites.

Digital technologies such as AirRater can play a role in public health responses to the growing challenges posed by environmental health hazards by (i) providing health departments and other agencies with an additional channel to communicate important environmental health information to populations, (ii) gaining population-level health data during serious environmental hazards, and (iii) directly supporting individuals by providing personalized environmental information that can inform behavior change leading to improved health outcomes. AirRater’s design allows it to fulfill all three of these functions.

The core logic underlying AirRater’s design is that by providing individuals with timely, local, easily accessible, and understandable environmental information in conjunction with the ability to link this information directly to personal symptoms, the app facilitates decision-making that protects health. In this way, AirRater extends beyond the information deficit model, recognizing that the provision of information is critical but often insufficient on its own to engender behavior change (e.g., [31,32]). As such, AirRater has dual objectives of supporting risk communication and sustained behavior change.

In addition to the functions that provide individual-level support, symptom data provided by registered users is aggregated and analyzed for the purposes of public health surveillance and epidemiological research. For example, strong associations between symptom reports and specific local allergenic plants have been identified through AirRater and used to provide locally relevant public information [29]. Given the app’s capacity to support both individual and public health outcomes, the Driving Force-Pressure-State-Exposure-Effect-Action (DPSEEA) framework represents a useful conceptual framework for the app (see Figure S3 in the Supplementary Material). The framework is commonly used to understand linkages between the natural environment and individual or public health outcomes [33,34] and demonstrates how AirRater can be utilized to support environmental health objectives. Specifically, the DPSEEA framework depicts AirRater’s role as an intervention designed to support individuals, the community and government agencies in achieving positive health outcomes in the face of environmental hazards in a changing climate. As an intervention, AirRater has the potential to support individual and population health outcomes at both the Exposure (as a prevention tool) and Effect (as an adaptation tool) stages of the DPSEEA framework.

### 1.2. Evaluation Process

Evaluation activities are used to establish the interaction between users and an intervention, providing an evidence base for the outcomes and impact of the intervention [35]. Evaluation of the app has been, and remains, imperative to ensure that it continues to meet its intended objectives as an environmental and public health tool, as well as meeting the needs of its stakeholders. The in-depth evaluation extends on previous user surveys conducted regularly to obtain feedback about functionality, usefulness and behavior change associated with use of the app. They have been conducted in a range of geographic locations and environmental conditions including the extensive and prolonged 2019–2020 bushfire season in Australia when the app uptake experienced considerable growth, with an additional 45,000 users downloading the app [28,30]. These surveys have consistently indicated the app has been used by individuals to support decision-making that protects health [28,30]. However, simple quantitative surveys are limited in the depth of information that can be collected and do not offer insights into the complexity of reasons behind the responses. This paper reports an in-depth, predominantly qualitative, evaluation of AirRater, including an investigation of user demographics and motivations of app use in different locations. Utilizing existing behavior change frameworks and models (discussed below) to interpret results, the evaluation contributes to the burgeoning literature on user perspectives of digital health interventions and their effectiveness in supporting users to achieve personal health outcomes.

A key objective of the evaluation was to investigate perceptions of the app’s usability and effectiveness to support decisions that protect health across three key stakeholder groups: existing users; relevant government agency and peak body representatives; and healthcare professionals. This paper presents results provided by AirRater users as the first key stakeholder group from which data were collected. The research aims were to:

(1) explore user perspectives on (a) AirRater’s usability as an intervention to help manage environmental health hazards, and (b) the app’s effectiveness as a tool to support decision-making and behaviors that protect health when exposed to a range of environmental hazards, such as bushfire smoke and high levels of pollen.
(2) to interpret the results in the context of two theoretical frameworks relevant to understanding and supporting behavior change—the Behavior Change Wheel (BCW) and the Protective Action Decision Model (PADM).

### 1.3. Behavior Change Frameworks

We selected two theoretical frameworks on behavior change to facilitate the theoretical triangulation of our data. The first framework, the BCW, was developed to support the implementation of effective behavior change interventions [36]. Beyond its rigor and validity, a key rationale for selecting the model was its use in the development and evaluation of numerous public health interventions (e.g., [37,38,39]). The BCW framework guides the development of effective interventions by encouraging implementation scientists to consider the broader context in which an intervention takes place. The central component of the BCW is the COM-B framework, which recognizes that individuals require capability, opportunity and motivation (i.e., COM) in order to achieve behavior change (i.e., B), and that these three essential conditions can interact with one another to impact an individual’s ability to achieve that change. The COM-B framework is “encased” by two outer layers. The first outer layer comprises nine intervention functions to assist in the identification of the most effective interventions. The second outer layer comprises seven policy areas to enable the implementation of the most effective interventions. Taken as a whole, the BCW guides the development of effective interventions by encouraging implementation scientists to first consider the behavioral conditions that need to be changed in order for an intervention to be most effective. While the BCW framework was not used during the development of AirRater, we have used the BCW framework to better understand how the app may support behavior change. Applying the framework to AirRater enabled us to characterize the app as a behavior change intervention in its present format and to identify potential enhancements to the app in light of the results.

The PADM provides theoretical insight for understanding individual responses to environmental hazards and disasters [40]. It is conceptualized as a feedback loop, commencing with cues or warnings (e.g., sights, smells, sounds, observation, information) that prompt pre-decisional processes in individuals. These pre-decisional processes can lead to perceptions in relation to the threat and possible protective action that can be taken. One of three behavioral responses generally ensues: (1) the search for additional information, (2) protective action, or (3) emotion-focused coping. Protective action will be taken based on an individual’s identification and assessment of personal risk and a scan of potential protective action options. These options are informed by the broader environmental and social context and are often temporary measures as opposed to permanent ones. The PADM also acknowledges that decision-making timeframes can be drastically shorter given decisions in relation to environmental hazards and disasters are often made following the unexpected onset of an emergency situation. Using the PADM framework supported the interpretation of data, particularly context-specific differences in use of the app in the face of different environmental hazards.

## 2. Methods

In the pursuit of gaining deeper insights into the personal and unique experiences of users, the epistemological and ontological underpinnings of this research align most closely with constructivist and interpretivist paradigms [41]. That is, users employ and access apps within broad social, cultural, organization, political, economic and environmental contexts. Accordingly, in an attempt to capture any location- and context-specific differences of app use, a predominantly qualitative approach was employed to collect user data for the evaluation [42]. A quantitative data collection method—a pre-interview questionnaire—was used to gather relevant demographic data, and a qualitative data collection approach—semi-structured interviews—was used to explore perceptions of usability and effectiveness with users. Further details of each component are provided below. To facilitate an independent evaluation of the app, the AirRater team approached a qualitative researcher (AW) not affiliated with any institutions or organizations involved in the design or development of the app to lead the evaluation. A framework and protocol were developed, with feedback provided by a second qualitative researcher who was not affiliated with the research team. The evaluation was approved by the University of Tasmania Health and Human Research Ethics Committee (ID: H0015006).

*AirRater characterization*. Using the BCW framework [36], one researcher (AW) initially identified functions contained in the app that may influence behavior change, and policy categories linked to the app that support delivery of AirRater across jurisdictions. 

*Participant selection*. We invited participants from three locations in Australia to understand whether location-specific differences and/or other factors influence the use of AirRater as well as personal perceptions regarding its usability and effectiveness:

1. Tasmania, Australia: AirRater has been deployed in Tasmania since 2015, with the Tasmanian Department of Health as a core funder since inception. Tasmania experienced severe bushfire events in 2016 and 2019, with prolonged poor air quality greatly impacting local communities. Tasmania also experiences high rates of allergic rhinitis and other allergies compared to other parts of Australia [43].
2. The ACT, Australia: AirRater was first deployed in the ACT in 2017 and ACT Health has been a core funder since then. ACT air quality was significantly impacted by an extensive bushfire event between October 2019 and February 2020; the app’s download rate in this location increased rapidly during that period. The ACT also has the highest allergic rhinitis rates in Australia [43].
3. Port Macquarie, New South Wales, Australia: AirRater was deployed in Port Macquarie in 2019 following a prolonged period of peat fire and bushfire events resulting in poor air quality.

*Participant recruitment*. Users were recruited using a convenience sampling strategy to investigate their perspectives of usability and the effectiveness of AirRater as a health protective intervention. Eligibility criteria stipulated users must be over the age of 18 and live in Tasmania, the ACT or Port Macquarie. Users located in Tasmania, the ACT and New South Wales who registered as AirRater study participants (*n* = 4336) were contacted via email in April 2020. A targeted follow-up email was sent to relevant individuals of this cohort (*n* = 2526) in order to recruit additional participants based in the ACT and Port Macquarie in May 2020. Concurrently, a targeted traditional and social media recruitment strategy was employed in an attempt to recruit additional Port Macquarie users. Additionally, recruitment information was included in an AirRater newsletter sent to subscribers (*n* = 1532) in May 2020. Bearing in mind timing and resource constraints for the evaluation, the protocol and consequent ethics application indicated up to 30 participants would be sought from each location. Ultimately, we recruited 42 participants from Tasmania (*n* = 20), the ACT (*n* = 21), and Port Macquarie (*n* = 1). To maintain participant anonymity, and given both locations experienced an episode of prolonged poor air quality, data captured in Port Macquarie have been aggregated with data captured in the ACT.

*Pre-interview questionnaire*. To acquire basic demographic data, participants were asked to complete an anonymous pre-interview questionnaire (see Table S1 in Supplementary Material) that was delivered via the online platform, SurveyMonkey. Data captured included gender, age range, number of dependents (children < 15 years old), residential location (by postcode), and income level. There were also some general questions about the app, such as primary motivation for downloading and self-perceived regularity of use over time. 38 of the 42 participants completed the pre-interview questionnaire and results were aggregated for descriptive reporting.

*Interview schedule*. An interview schedule was developed to guide conversations [44]. This involved identifying six overarching themes considered pertinent to explore with participants, informed by the needs of funders and AirRater team members. The overarching themes were: (i) use of AirRater; (ii) features of AirRater; (iii) comprehension of and trust in AirRater; (iv) self-management and AirRater; (v) behavior change and AirRater; and (vi) translational capacity of AirRater (see Table S3 in Supplementary Material).

*Semi-structured interviews*. Semi-structured interviews were carried out in May and June 2020 by one researcher (AW). Written consent was received from each participant to record the interview. All interviews were carried out via phone or virtual communication platform based on a participant’s preference. The average length of an interview was 32 min.

*Data analysis*. All interviews were recorded and transcribed verbatim by one researcher (AW) and one administrative AirRater team member. Each transcription was verified for accuracy by one researcher (AW) prior to analysis. The six overarching themes that comprised the interview schedule provided the framework for thematic analysis of the data [45], with the six themes used as an initial coding structure that supported the identification of sub-themes as they emerged from the data. All transcripts were imported into the qualitative analysis software, NVivo 12 [46]. Coding and analysis of the qualitative data were initially undertaken by one researcher (AW). Results were discussed with three other researchers (PJJ, AJW, SLC). Data were triangulated with previous AirRater survey results and through the use of two theoretical frameworks (BCW and PADM).

## 3. Results

Results are presented as follows: (i) AirRater characterization, (ii) participant characteristics, (iii) AirRater use, (iv) self-management, (v) behavior change, and (vi) capacity to share information provided by AirRater with others. For an overall summary of the results, see Table 1.

### 3.1. AirRater Characterization

We used the BCW framework to better understand how the app may support behavior change. In applying the framework, we identified that the app currently utilizes four interventions to support behavior change:

(i) Education (through in-app links to further information on the AirRater website);
(ii) Persuasion (through the color-coded rating system);
(iii) Environmental restructuring (through notifications and alerts); and
(iv) Enablement (through the symptom and environmental hazard summary feature) (see Table S3 in Supplementary Material; refer to Table S4 in Supplementary Material for relevant definitions).

Using the BCW framework, we also determined that population health data collected by AirRater informs a number of policy categories for government health and environment agencies involved in the collaboration. Specifically, AirRater data informs the development and implementation of:

(i) Comms/marketing (through media campaigns);
(ii) Guidelines and
(iii) Regulation (included in guidance to the public provided by health agencies); and
(iv) Environmental/social planning (informing the creation of shelters and decision-making around the monitoring network) (see Table S3 in Supplementary Material; refer to Table S4 in Supplementary Material for relevant definitions).

### 3.2. Participant Characteristics

In total, 38 of the 42 participants completed the pre-interview questionnaire (see Table S5 in Supplementary Material for a summary of responses). Over half (*n* = 21) began using AirRater in the past year. The majority of participants (*n* = 30) identified as female. Age range distribution of participants was roughly equal across most decadal age ranges, with slightly lower representation in the 21–30 and 41–50 age range brackets. Over half of the participants (*n* = 24) indicated they experience allergic rhinitis, with some (*n* = 17) indicating they experience asthma. Two users indicated they live with diabetes and three users indicated they have a heart condition. Approximately a third of users (*n* = 12) indicated they experience other conditions, including allergies and anaphylaxis to foods and other products, rheumatoid arthritis, auto-immune disease, chronic renal disease and hypertension. A majority of participants (*n* = 33) reported having no children under the age of 15 years old. Personal income distribution varied between participants, with approximately one quarter of users (*n* = 11) reporting a personal annual income bracket of less than AUD$41,599.

### 3.3. AirRater Use

Participants were asked when they downloaded the app and why. They were also asked how they found out about the app, whether there were any regular patterns of use, their motivations and prompts to use the app, and whether their use had changed over time. Many participants indicated their experience with a particular health condition, such as allergic rhinitis or asthma prompted their decision to download the app. For other users, curiosity or a desire to contribute to research was the primary motivation:

‘I don’t really have particular symptoms…I’m interested in science so it appealed to me, and the fact that we live in [location] and I knew that there were air quality issues in [location], so those things all added up probably.’

(ID_27, TAS)

After downloading the app, participants identified numerous prompts and motivations for checking the app. Environmental visuals regularly motivated a small number of users to check the app, while the symptom reporting push notification functionality of the app acted as a prompt for others. Many participants indicated that experiencing symptoms acted as a prompt:

‘I think sometimes I would check the app if I thought my symptoms were getting worse just to check if there was a reason for that, or conversely if they were getting better, so I guess my symptoms were something of a trigger.’

(ID_10, ACT)

A number of participants discussed how they would use the app to validate or verify physical responses to environmental hazards:

‘I’d feel bad and I’d think, what’s the air quality today, and then I’d check, and you know, nine times out of 10 the air quality would be bad and that’d make me feel better because I’d think, oh I’m not getting sick, it’s just that it’s bad air quality, I’ve got a bit of asthma.’

(ID_6, TAS)

A few participants indicated that they proactively used the app to monitor air quality or pollen levels in locations to which they were travelling, or to monitor locations where family members were based:

‘…I could…monitor not just how we were going, but them [family], and use AirRater to determine whether they were likely to be having a good day or bad day and whether to give them a call or, you know, if they would be OK today.’

(ID_10, ACT)

Many users indicated that the app was useful, straightforward and user-friendly. Some participants indicated that the app not only provided them with the information they needed to implement behaviors that protect health, but also enabled them to easily communicate information to others, including family and community members:

‘…I don’t want to trivialize the amount of work that must go into keeping it going, but from a user’s perspective, it’s got a simple function, and it’s simple to use, and it reports simple information…and that’s probably one of its biggest strengths. I can get the information I need quickly, without having to really do anything.’

(ID_30, TAS)

‘…the other thing that I found really useful with it was because other people don’t have the same reactions to smoke…I was able to give them a visual. I was able to turn the phone around and say, look, this is the level at which it is in this current location.’

(ID_34, ACT)

Numerous users who downloaded the app in the ACT during the prolonged smoke event between October 2019 and February 2020 indicated that using the app during that period led to both physical and psychological benefits:

‘it has both a…direct biological health impact for me…but it also has this psychological soothing aspect, side-effect…that I personally found extraordinarily beneficial, and that I think other people will, given the uncertainty of the sort of world that we’re living in.’

(ID_14, ACT)

‘…I found having the app, for me having more information is calming. It, you know, helps, with the anxiety, so for me, having that, instant feedback of this is what it’s been like and the reading is no more than an hour old is really useful.’

(ID_34, ACT)

### 3.4. Self-Management

Participants were asked whether the app provided them with information to assist in self-management of any symptoms, specifically with the use of medication. Participants provided varied responses in relation to the app’s capacity to support them with self-management. A number of users indicated the app assisted them to self-manage in relation to medication use:

‘…I’d look at the app and be like, oh I actually haven’t taken Ventolin today…So it became a ritual for me…while I was really symptomatic…it helped me form a schedule…of using my medication.’

(ID_14, ACT)

‘I think because it prompted me to put my symptoms in, I actually was a bit more aware about what was happening, whereas if I hadn’t been doing that, it might have taken me a bit longer to go, I need to increase my preventers, just because life’s busy and you’re not thinking about that.’

(ID_31, ACT)

A small number of users indicated that using the app would remind them to carry medication with them:

‘…if I was going into campus and it said that there were high levels…then I would perhaps think about using, taking medications with me so I can use them during the day and things like that.’

(ID_28, TAS)

A number of users also indicated that through using the app, they came to know their own personal exposure thresholds:

‘…it’s led me to understand a lot about myself and the symptoms that I have. I really hadn’t realized that I had…such bad allergies and hay fever problems.’

(ID_3, TAS)

‘…given our experience with the smoke, that overnight usually it came in…when the wind dropped, and you’d wake up in the morning and you couldn’t see a foot in front of yourself…understanding that that was occurring, helped me to actually manage the way I took my medication…’

(ID_37, ACT)

Half of the users, however, indicated that the app did not directly support them in self-management, as their use of medication was dictated by the symptoms they experienced or they used preventive medication to avoid symptoms in the first place. In particular, users who had chronic conditions or well-established management plans indicated that the app didn’t support their routine self-management:

‘No, my symptom management is…medication driven and experience driven…if I get up and I’m sneezing and streaming…I don’t need the app, I take an antihistamine. And I’m on medication for the asthma…the app’s not relevant in that respect.’

(ID_29, TAS)

‘…as soon as…the pollen season…is coming up, I’ll try to be taking them [medication] before…I’m exposed to the pollen…I find they’re just not effective otherwise.’

(ID_41, ACT)

### 3.5. Behavior Change

Participants were asked whether AirRater provided them with information to assist in decision-making for their day. A majority of participants indicated that information provided by AirRater had the potential to influence their behavior, with participants demonstrating both proactive and reactive behavior change:

‘…if the particulate rating was high…we definitely changed our plans according to that information. So, we would not go to the shops or not go out for a walk or…advise our kids to make sure that they knew that…it was pretty bad out there.’

(ID_10, ACT)

‘…one of my colleagues…suffers quite badly from hay fever…he uses it to check what’s going on when he feels symptoms, whereas I use it to prevent symptoms…the pin drop is very useful for me because it allows me to point it to where I’m going, not where I am, to work out what might need to happen…’

(ID_30, TAS)

‘…when I saw that the air quality was pretty good, I just carried on walking…it’s a sort of security blanket…it just reassures you that you can keep on chugging along.’

(ID_18, TAS)

‘I…put in three locations that I use all the time…one’s the Australian National Botanic Gardens where I’m a volunteer guide, and particularly during the bushfire it was important for me to know what was happening over there, because if…it was bad I wasn’t going to go and volunteer basically.’

(ID_4, ACT)

One participant indicated that they thought the app assisted individuals in making decisions more quickly during a stressful or uncertain situation:

‘…1994 I think, when we were living in Sydney the bushfires just stopped at our doorstep…we had to make a decision whether to stay or to leave…it was very hard to make that decision…and ultimately…we did leave…because of my asthma…if I’d have had that app at that point in time, we’d have made that decision earlier….this is the most important aspect of this app…it will actually help people make decisions to stay or to go, and make them earlier and more intelligent and sensible…’

(ID_18, TAS)

A number of participants indicated that they would not change their behavior as a result of the information the app provided:

‘…the fact that I usually work on campus, so it’s not like I have a choice, yeah, and I usually prefer seeing people face to face anyway. So, even if I have the choice to work from home, I’d prefer not to…’

(ID_2, TAS)

‘…my day has got to be the way it is. If I have to go out, I have to go out…when I have to go out, that’s it, it doesn’t matter what the conditions are like and I’ve done some driving…in conditions that made my hair turn white, but I still had to do it.’

(ID_29, TAS)

‘Not really, you know, just life, get on with it…we might have dried our clothes indoors rather than outdoors…that was about it.’

(ID_5, ACT)

‘I was at the point where I was thinking that I need to get out of Canberra, it was that bad. But then once you start looking what was happening elsewhere, I didn’t quite know where I was going anyway, and I was physically not able.’

(ID_37, ACT)

Other participants indicated they could not change their behavior, but would modify it where possible to minimize potential health risks

‘The other thing is that I tended to ride to work, it’s not far, it’s only 5k [kilometers] ride, pretty flat, but I would wear a mask to and from work…so I would certainly check before I got on my bike, but realistically I’m getting on my bike and it’s going to be bad.’

(ID_36, ACT)

‘…I’d be more careful about exercising outside, I ride a bicycle in and out of work, so I might be more careful about riding a bit slowly, and not pushing myself too hard…if I was going to go for a walk or something at lunchtime and the air quality was bad, I might rethink that.’

(ID_6, TAS)

Some participants based in the ACT indicated that beyond the individual level, the app supported decision-making at an institutional and operational level:

‘…that first Saturday we turned up at cricket and we’re going, you know, they said, you know, the game can proceed if the number is less than whatever the number was, how do we do that? I…pulled my phone out…pulled up AirRater and went, there’s the answer.’

(ID_5, ACT)

‘…we’ve got a number of sites in the department I work for…and we had varying…air quality across our different campuses, so…it was good for me to have that just in my pocket so I could look at it and work out if we were going to have an issue with one of our campuses or not.’

(ID_42, ACT)

Further, users in ACT indicated a variety of ways AirRater was used to support behavior change during the bushfire smoke event. Examples ranged from making behavior changes around the home, to exercising, to informing decisions to relocate in the face of increased risk of health complications:

‘…it also helped us…realize how much we needed to…make sure that the house was smoke-proof…it wasn’t just a matter of staying inside, it was actually a matter of making the inside space better.’

(ID_34, ACT)

‘The other thing that was really helpful for me…is that I have evaporative cooling. …But the problem with evaporative is that you have to have the windows open…it really helped me…to know when not to put my evaporative cooler on…it was really valuable.’

(ID_42, ACT)

‘…before breakfast I always go for…a walk…and it was important for me to know if it was safe for me to do that or whether I was going to have to go to the gym and do it on a treadmill…’

(ID_4, ACT)

‘I was using it as a method…to try and encourage them to get out of their house…because the smoke was getting so bad…I was sending screenshots of the app to my Dad…I annoyed them enough into coming and it was good for them as well, because my Mum also has lung issues…’

(ID_14, ACT)

### 3.6. Capacity to Share Information Provided by AirRater with Others

Participants were asked whether they had discussed AirRater with two groups: (1) family, friends and/or colleagues; and (2) healthcare professionals. Participants were also asked their perspective on the ability of AirRater in its current form to support individual health, particularly at the patient-doctor interface. A majority of participants indicated that they had spoken with family, friends and/or colleagues about the app since downloading and using it, particularly those suffering from a respiratory condition or symptoms:

‘…I recommended it to everyone. You need this, now.’

(ID_34, ACT)

‘We encouraged our students to use it so they could keep track of what they were experiencing. And I think they also found that really sort of rewarding because…it was such a…traumatizing time…’

(ID_14, ACT)

‘Ah yes, any of my friends, I’m of a certain demographic, I’m 65, so a lot of my friends are starting to have lung problems...’

(ID_24, TAS)

Approximately one quarter of participants indicated that they had spoken with their doctor about AirRater:

‘…I’ve mentioned AirRater to my doctor and that I use it, and that’s part of my asthma plan.’

(ID_24, TAS)

‘…I went to the doctor last December…I just said, look I’m not controlling…my coughing…and I said, on this app it says…and she went, what app? And I said, oh it’s an app the ACT government is doing in conjunction with Tasmania and they’ve bought it so Canberrans can use it. Anyway, the doctor downloaded it then and there...’

(ID_38, ACT)

One participant with a medical background expressed their views on the app’s capacity to support public health:

‘I think because it gives you good outcomes, and everybody who actually uses it…properly can actually see that…it’s working very well…all we’re doing is we’re trying bit by bit, by word of mouth, to increase the cohort of people who have an engagement with it, cos it is useful but you have to put the effort into it.’

(ID_3, TAS)

Several participants noted that heightened healthcare awareness of the app would be beneficial:

‘I think that more medical professionals should be made aware of the AirRater app. The doctors, I go to a practice with several doctors, and sometimes you just get who’s there, none of them were aware of it…They thought it was a great idea, but they weren’t aware of it.’

(ID_24, TAS)

## 4. Discussion

Here, we contextualize our results using the BCW and PADM frameworks, along with the existing literature, including previous AirRater user survey results. We then discuss limitations associated with the user evaluation. Next, we consider future opportunities for AirRater as well as the implications of our results for future research. We conclude with some remarks on the utility of the frameworks used to aid in the interpretation of our results.

### 4.1. AirRater in the Context of Existing Frameworks and Literature

Overall, our results from participating users indicated that the app has the capacity to support decision-making that protects health. Characterizing AirRater using the BCW framework elucidated that AirRater employs four interventions that engage five of the six conditions Michie et al. consider essential for behavior change (all except automatic motivation; see Table S3 in Supplementary Material) [36]. Through its design, the app endeavors to support behavior change by increasing knowledge and understanding of environmental hazards through communicating information using words and colors to elicit a response and/or action (persuasion); changing the social context by enabling engagement with environmental hazards through various traditional and online media, including Facebook and Twitter (environmental restructuring); and increasing users’ capacity to implement behaviors that protect health by supporting users to understand their own personal exposure thresholds (enablement). At the policy level, population health data collected via the app are utilized by AirRater collaborators with policy-making capacity, predominantly at the subnational level. Collaborators use data provided by AirRater to inform the development and implementation of air quality standards, guidelines and regulations; the promotion of AirRater on their website (comms/marketing); as well as the creation of shelters and decision-making around the monitoring network (environmental/social planning).

Beyond validating the utility of the app, it is useful to consider how results from the user evaluation compare with conclusions drawn in the existing literature. Our results show that the majority of participants live with at least one pre-existing condition and use the app to implement behaviors to protect health. This finding is congruent with the conclusions of both previous AirRater user surveys [28,30] and other studies. For example, in their exploratory study of health protective behavior in 842 participants, Harris and Guten (1979) found that those who identified as being in poor health were more likely to avoid pollution than those who identified as being in moderate health or good health [47]. This has been confirmed more recently in a systematic review of adherence to air quality information. D’Antoni et al. (2017) identified numerous studies that concluded individuals with a pre-existing health condition demonstrated significantly higher rates of adherence to air quality-related health advice compared with healthy participants [48]. We also found just under half of participants were prompted to use the app following the experience of personal symptoms, with smaller numbers prompted by reminders and environmental visuals. These results align with findings in the existing literature on responses to environmental hazards. Results from a study of citizens in Birmingham, United Kingdom (UK) found that personal health impacts and sensory cues were avenues for raising awareness of air pollution [24]. Further, D’Antoni et al.’s (2017) review found that the experience of personal symptoms was a common predictor of behavior change to protect health. Our results add to the existing literature on the role that digital health interventions can play in supporting vulnerable population groups to manage their health.

Results relating to the app’s capacity to assist users with self-management, specifically with their use of medication, were mixed. Just under half of participants reported the app assisted them with medication use. The app also provided a number of participants with greater awareness about the impacts of exposure to an environmental hazard on their individual health outcomes. Just over half of participants do not use the app to support their medication use, instead relying on the experience of symptoms to prompt medication use and management or taking medication to avoid experiencing symptoms. Results relating to behavior change demonstrate that certain barriers challenge sustained behavior change that protects individuals from exposure to environmental hazards. Participants indicated these were often driven by practical considerations, such as work commitments. Haddad and de Nazelle (2018) found similar results in their study on the influence of smartphone technology on travel behavior change, with time and practical considerations influencing the capacity for participants to choose health protective travel routes [49].

The evaluation results complement and extend findings in earlier AirRater user surveys [28,30]. There was congruence between previous survey data and our evaluation data on perceptions of AirRater as a useful, easy to navigate and user-friendly app that enabled respondents and participants to make decisions and change their behavior in the face of environmental hazards. Similarly, as previously reported in user surveys, a majority of evaluation participants use the app personally, as opposed to someone they care for. The evaluation extends our understanding of how AirRater is used by providing further insights into motivations for use, including initial motivations for downloading the app as well as symptom and environmental prompts for app use over time. The evaluation also provided greater insights into the varying ways and extent to which participants used the app to support self-management through medication use and shared information from the app with others.

Moreover, the evaluation results facilitated comparison of app use in different locations and under different environmental conditions. ACT participants, who were impacted by the 2019–20 bushfire smoke event, provided a number of distinct responses compared with their Tasmanian counterparts. For example, while both Tasmanian and ACT users acknowledged the app verified and validated their physical symptoms, ACT users asserted that the app provided psychological benefits in the face of a natural disaster. ACT users also discussed using the app beyond the individual level to support decision-making at an institutional or operational level during the bushfire smoke event.

Results from participants who were based in the ACT during the bushfire smoke event align particularly well with the theoretical tenets of PADM [40]. Most users in the ACT downloaded the app following the arrival of bushfire smoke (an environmental hazard cue) and were motivated to use the app to prevent experiencing symptoms (physical cue) or for safety (protective action). Specifically, ACT users most commonly utilized the app to inform their decision making around whether it was safe to go outside or whether to stay inside. Finally, a number of ACT users articulated using the app to facilitate emotion-focused coping as a behavioral response to the bushfire smoke, acknowledging that app use provided a psychological benefit at a particularly stressful time.

### 4.2. Limitations

It is important to note limitations of the study design. Firstly, we acknowledge that the findings of this small qualitative study are not generalizable to the wider population in a quantitative way. We note that rigor in qualitative research does not depend on the number of participants [50]. This is because the primary purpose of such research is to gain in-depth understandings of the breadth of experiences of participants, rather than the collection of representative population data. Our results added considerable depth to the findings from previous quantitative participant surveys and have provided new insights into motivations for behavior change, which will be used to improve the app to further support decision making to protect health in the face of changing environmental conditions. Secondly, limitations in relation to data analysis need to be acknowledged. Given the research team’s desire for an independent evaluation, one researcher (AW) who was not affiliated with the design and development of the app was responsible for data collection and analysis. While efforts were pursued to limit the potential for researcher bias and/or error through the development of a comprehensive study framework and protocol, this limitation should be kept in mind when considering the results presented above.

A number of additional limitations should be acknowledged in relation to the evaluation itself. Firstly, several avenues were pursued to specifically recruit users from Port Macquarie, including recruitment emails to users, social media posts and a local radio interview. Despite these attempts, only one participant was successfully recruited for Port Macquarie. Acquiring additional perspectives from Port Macquarie users would have enabled a more meaningful comparison of perceptions of app usability and effectiveness for bushfire smoke as an environmental hazard across two different locations (the ACT and Port Macquarie). Secondly, for the small number of users who had downloaded the app in the three months prior to interview, mobility restrictions associated with COVID-19 impacted their ability to speak to certain features and functionalities, such as the utility of the map function. Given a majority (80%) of participants had downloaded the app more than six months prior to the evaluation, the consequences of this limitation on findings are likely to be minimal. Thirdly, not all population groups participated in the evaluation. While the age range of participants was relatively equally disbursed across most decadal age ranges, there were no participants under the age of 21. Similarly, the evaluation did not capture data from any parents who use the app specifically for the care of a dependent.

In addition, there was also no representation from culturally and linguistically diverse (CALD) individuals. Brewer et al. (2020) warn against the perpetuation of health inequities through the development of digital health interventions lacking sufficient input from, and consideration of, racially and ethnically diverse communities [51]. While a key objective of AirRater is to address health inequities through the provision of free and easily accessible information, targeted efforts should be employed to seek feedback specifically from CALD individuals in future user surveys and evaluations. Given AirRater is only available in the English language, future research should investigate whether this impacts its utility and uptake among CALD individuals. Finally, participant recall bias is a possibility, particularly for those who used the app during stressful periods, such as the bushfire event. Given our objective was to capture perceptions of app usability and effectiveness based on self-reported experiences, we do not view this potential limitation as impacting the validity of the data or our findings.

### 4.3. Future Opportunities for AirRater and Implications for Future Research and Practice

Considering our results in the context of the BCW framework has identified opportunities that may enhance the effectiveness of AirRater as an intervention. Specifically, there are two additional intervention functions could be incorporated into the app to enhance its utility: incentivization (introduction of a reward mechanism to further encourage use) and training (skill development by highlighting specific actions that can be taken to reduce environmental hazard exposure).

To elaborate further on these opportunities, the app is currently limited in its capacity to support user customization and does not explicitly provide corresponding, tailored health advice with its color-coded word rating system. D’Antoni et al. (2019) undertook a randomized controlled trial investigating the impact of alternative delivery avenues of air quality information, the specificity of that information, and behavior change. They determined that using messages delivered through a smartphone app that provided specific, customized behavioral guidance led to substantially more respondents indicating a commitment to health protective behaviors compared to those who received official government air quality information [52]. Mehiriz and Gosselin (2019) documented similar findings in their randomized controlled trial investigating the effect of a smog warning alert system on behavior change in Canada. In their study, the treatment group (i.e., those who received a warning with corresponding health advice) was more likely to undertake health protective behaviors than the control group [53].

In conjunction with supporting a level of personal customization, there are opportunities to enhance the capacity for users to share information provided by the app with others. Specifically, introducing a mechanism that enables users to more conveniently share their personal symptom data with healthcare professionals may encourage sustained engagement with the app and facilitate beneficial conversations at the patient-clinician interface. In their systematic review of asthma self-management, Hui et al. (2017) found that interventions that enabled feedback as well as decision support for healthcare professionals improved both asthma control and quality of life for patients [54]. Participants suggested further efforts to actively engage with healthcare professionals exist and should be pursued in order to expand the impact and reach of the app. Digital health interventions and monitoring platforms are becoming increasingly integrated into the healthcare setting [55], buoyed by both national and state commitment to the implementation of an Australian digital health strategy and framework for action [56,57]. In this enabling environment, pursuing strategies to engage healthcare professionals and policy makers is likely to further AirRater’s integration into the healthcare setting [58].

With these opportunities in mind, it is useful to note implications for future research. To comprehensively evaluate the utility and effectiveness of AirRater as an intervention, we will undertake a second phase of the evaluation. The second phase will involve semi-structured interviews with additional key stakeholders, including relevant government agency and peak body representatives, as well as healthcare professionals, in order to investigate knowledge, attitudes and practices regarding digital health interventions such as AirRater. Undertaking this component of the evaluation will assist us to validate AirRater’s current and potential role as a public health tool to understand population-level impacts of exposures to environmental hazards, as documented in the DPSEEA framework (see Figure S3 in Supplementary Material). Data from all stakeholders will ultimately inform future iterations of the app in order to optimize its utility and effectiveness, and to further support its integration into the healthcare setting. We anticipate results from the second phase of the evaluation will reveal important implications for future policy and practice, in keeping with recommendations for enhanced air quality research and communications by the Australian Royal Commission into National Natural Disaster Arrangements following the extreme bushfires and protracted severe air pollution during the summer of 2019–2020 [59].

Finally, when considering implications for the evaluation of digital health interventions more broadly, the findings presented in this paper meaningfully contribute to the existing literature that evaluates the effectiveness of digital health interventions to support the achievement of positive individual and community health outcomes. Beyond assisting us to identify the strengths of AirRater and opportunities for enhancement, utilizing behavioral change frameworks and models (BCW and PADM) provided a comprehensive and rigorous strategy for evaluating the mechanisms of a digital health intervention. A plethora of smartphone health applications are now available for consumers, yet very few include an evidence-based approach to behavior change [60]. Using the BCW framework and PADM model was a novel and successful approach to evaluate the AirRater app’s capacity to support participants to make decisions that protect health in the face of various environmental hazards. This approach could be utilized by designers and evaluators of other environmental health apps in future.

## 5. Conclusions

With environmental hazards likely to increase with a changing climate, the importance of effective interventions that successfully support users to undertake behaviors that protect health is imperative. As an app that acts as both a prevention and adaptation tool, AirRater provides an example of how apps can be utilized to support individual and community health. User perspectives on AirRater’s usability and effectiveness confirm that the app can increase individual awareness about the impacts of exposure to environmental hazards and provide useful information that supports users to self-manage and implement behaviors that protect their health in the face of environmental hazards. Using the BCW and PADM frameworks to interpret our results reaffirmed and extended upon findings in previous AirRater user surveys that the app is a useful tool that supports decision-making to protect health. Using these frameworks also allowed us to pinpoint specific interventions and policies currently embedded in the app to support behavior change. Further, participant data in conjunction with the BCW framework facilitated the identification of opportunities to enhance the effectiveness of the app for users, and further embed the app into the healthcare setting. These include supporting user customization and introducing a mechanism to more conveniently share personal symptom data with healthcare professionals. A number of study limitations were identified, which impacts the generalizability of results beyond the populations studied. Despite these limiations, the results enabled us to gain new insights into motivations for behavior change, which will be used to improve the app to further support decision-making to protect health in the face of changing environmental conditions.

## Figures and Tables

**Table 1:** Summary of key results from interviews with AirRater users, stratified by location.

Theme	Tasmania Users (*n* = 20)	ACT and Port Macquarie Users (*n* = 22)
AirRater use	Downloaded AirRater: Ranged from 3 months to 5 years Motivations for use: When (person caring for) experiencing symptoms Visual prompts, e.g., smoke Symptom reporting prompt	Downloaded AirRater: Since August 2019 Motivations for use: When experiencing symptoms Visual prompts, e.g., smoke Symptom reporting prompt To prevent experiencing symptoms/for safety
Self-management	No medication use/app does not assist with medication use Initiated/supported medication usage Carrying medication when travelling/ outdoors	No medication use/app does not assist with medication use Initiated/supported medication usage
Behavior change	Safe to go outside or stay inside Close windows Washing inside Air con use Avoid particular locations where there are triggers No behavior change Whether to exercise/play sport/garden Continue exercising Someone else mows lawns Change work plans	Safe to go outside or stay inside Close/seal windows Washing inside Air con use Avoid particular locations where there are triggers No behavior change Use P2 mask Whether to exercise/ play sport/volunteer/garden Relocation during bushfire Cleaning air con filters Working from home
Capacity to share information with others	Discussed with/recommended to family Discussed with/recommended to friends Discussed with/recommended to colleagues Discussed with/recommended to clients/students Did not discuss with doctor Discussed with healthcare professional Recommended by doctor	Discussed with/recommended to family Discussed with/recommended to friends Discussed with/recommended to colleagues Discussed with/recommended to clients/students Did not discuss with doctor Discussed with healthcare professional Recommended by doctor

## Data Availability

Requests for access to de-identified data presented in this study can be made to the corresponding author.

## Supplementary Materials

The following are available online at https://www.mdpi.com/article/10.3390/ijerph18073591/s1, Figure S1: Graphics of the AirRater app, demonstrating key functionalities. Figure S2: Schematic representation of the AirRater platform. Figure S3: DPSEEA framework. Table S1: Pre-interview questionnaire. Table S2: Interview schedule for semi-structured interviews with AirRater users. Table S3: Characterization of AirRater using the BCW framework. Table S4: Definitions for model of behavior, intervention functions and policy categories for the BCW framework. Table S5: Summary of participant characteristics stratified by question.
