# Supplementary material for: Environmental Hazards and Behavior Change: User Perspectives on the Usability and Effectiveness of the AirRater Smartphone App
Source: Int J Environ Res Public Health. 2021 Mar 30;18(7):3591. doi: 10.3390/ijerph18073591 (PMC8036707; doi:10.3390/ijerph18073591)
Supplement: Supplementary file 1 [file ijerph-18-03591-s001.pdf]

Supplementary Material

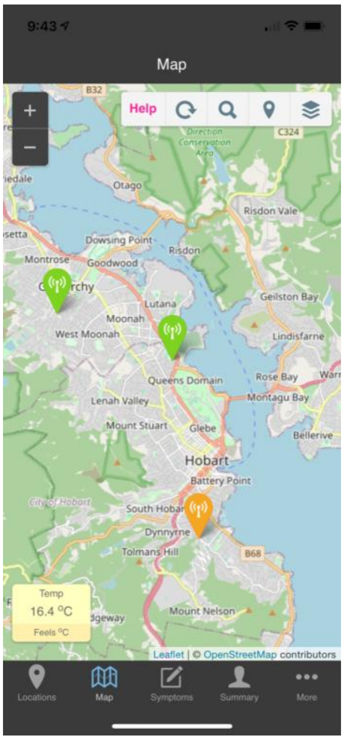

i) Map functionality with monitoring station overlay

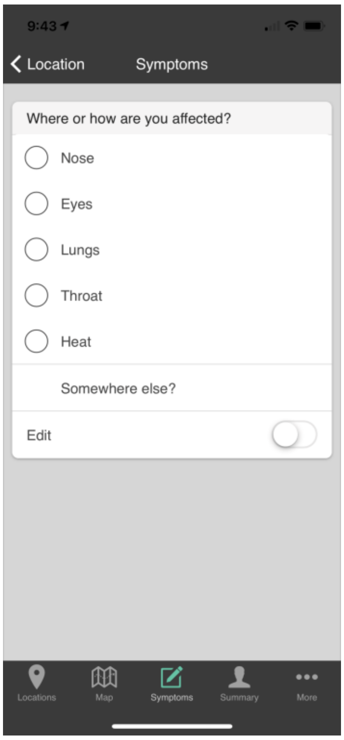

ii) Symptom reporting functionality

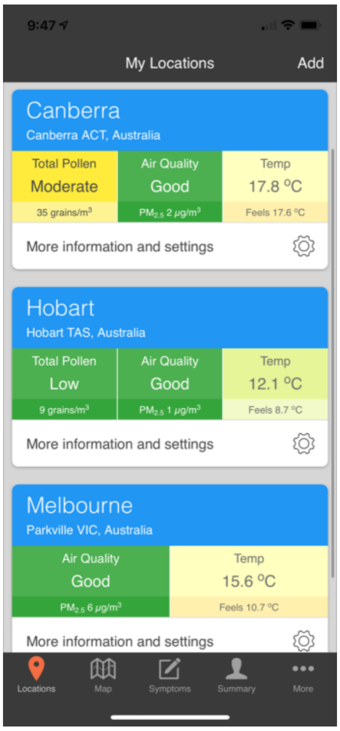

iii) Location functionality

Figure S1. Screenshots of the AirRater app, demonstrating map, symptom reporting and location functionalities

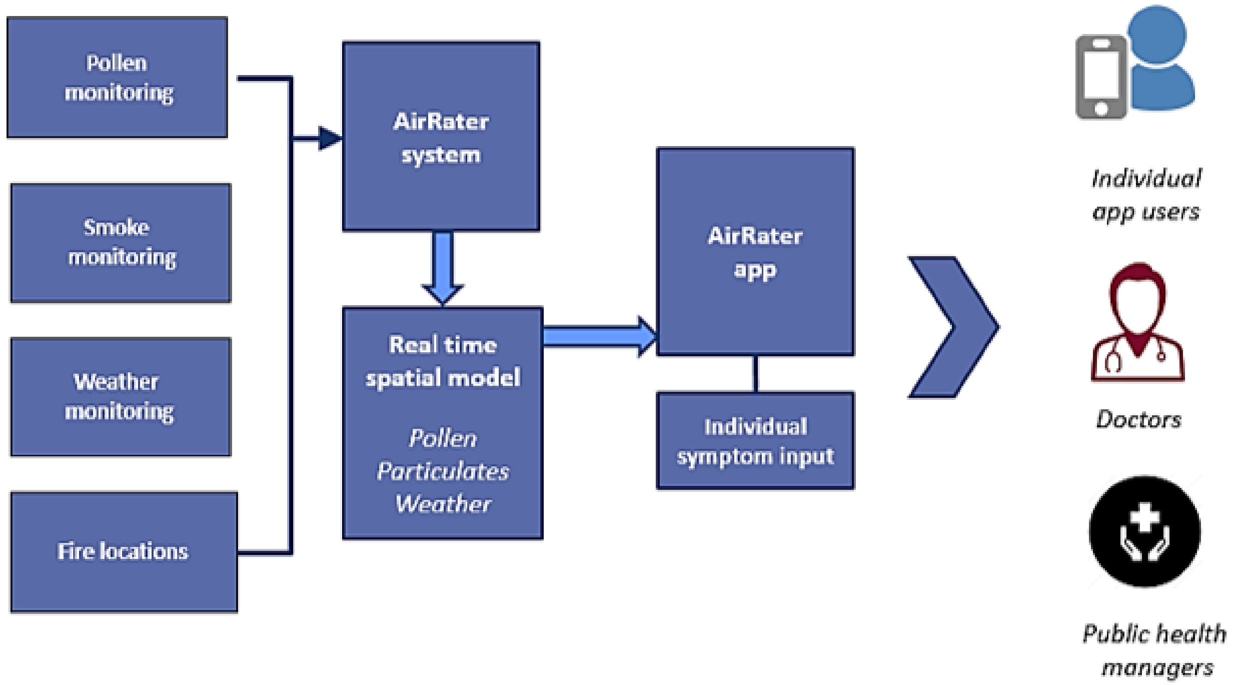

Figure S2. Schematic representation of the AirRater platform. Modified from Johnston et al. (2018).

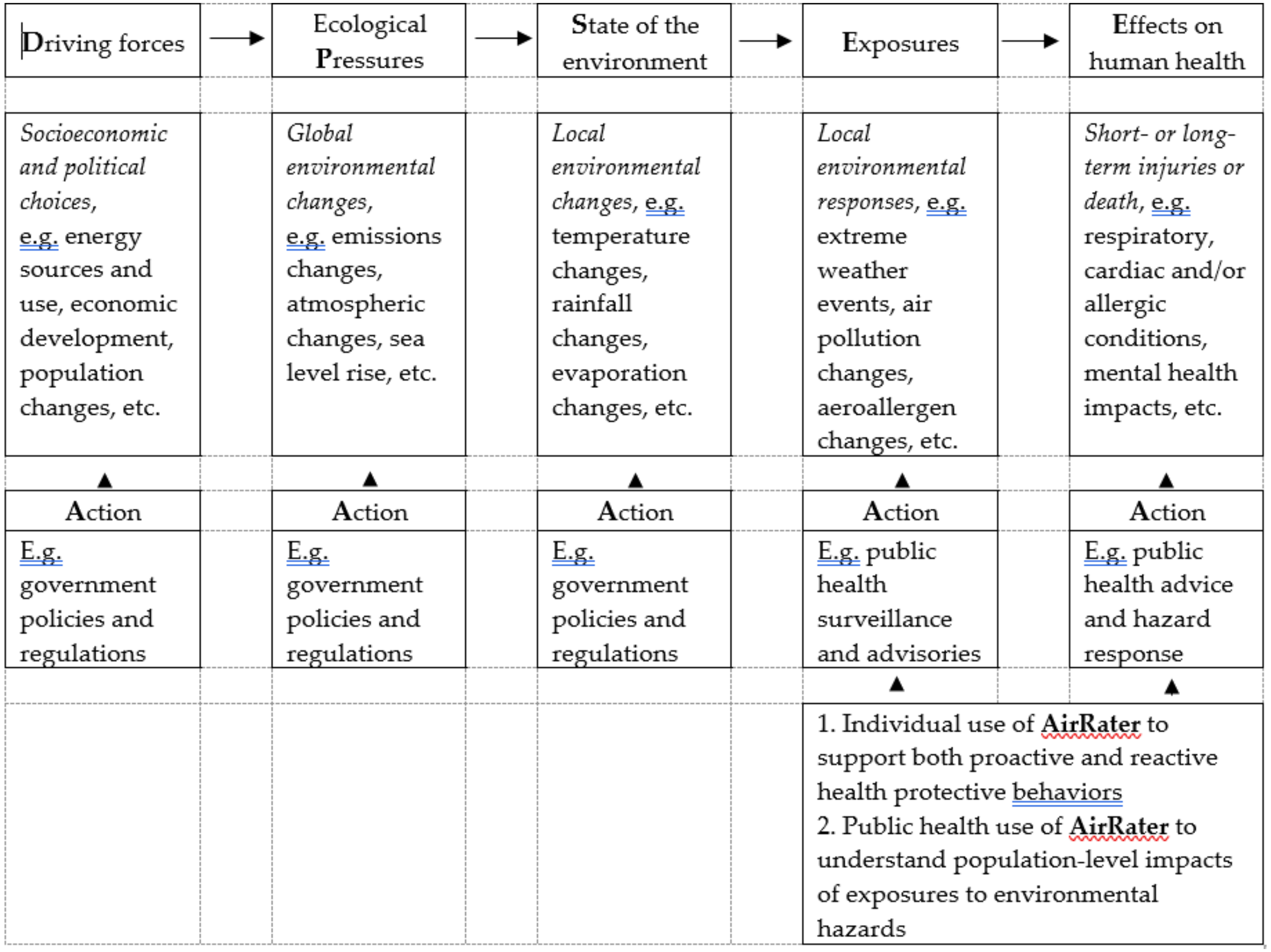

Figure S3. DPSEEA framework, modified from Boylan et al. (2018). The framework demonstrates AirRater's role as an intervention that supports individual and community health outcomes as both a prevention and adaptation tool.

Table S1. Pre-interview questionnaire

**Question No.**	**Question**	**Response Options**
Opening statement	Thank you for agreeing to take part in the evaluation of AirRater. Before you tell us about your experiences with AirRater in more detail in an interview or focus group discussion, it is important to know some information about you as a user and to gain a basic understanding of your thoughts about AirRater.<br><br>Below are some general questions about you. By answering the questions below, you will help us to better understand the different users of AirRater. The survey should take approximately 10 minutes to complete.<br><br>Your privacy is important to us. The information you provide below is anonymous. Any information you provide that we use for reporting will be grouped with information provided by all respondents so that you are not identifiable.<br><br>If you have any questions about this survey or for more information, please contact the AirRater team at <air.rater@utas.edu.au> or call 1800 322 102.	n/a
Your thoughts about AirRater	Q1. In your mind, what is the purpose of AirRater? (How would you describe AirRater to a friend?)	{Open text response}
	Q2. How did you find out about AirRater?	{Open text response}
	Q3. What was the main reason you decided to download AirRater?	{Open text response}
	Q4. Do you use AirRater for yourself, or do you use it for someone you care for? (e.g. a child with asthma)? (Please select one option only)	a. For myself<br>b. For someone I care for<br>c. I use it for my myself and for someone I care for
	Q5. Please provide a brief indication of how regularly you use AirRater.	{Open text response}

	Q6. Approximately how long ago did you download AirRater? (Please select one option only)	a. Less than 6 months ago<br>b. 6–12 months ago<br>c. 1–2 years ago<br>d. 2–3 years ago<br>e. 3–4 years ago<br>f. 4–5 years ago
Information about you	Q7. What is your gender? (Please select one option only)	a. Male<br>b. Female<br>c. Other<br>d. Prefer not to say
	Q8. What is your age range? (Please select one option only)	a.18–20<br>b. 21–30<br>c. 31–40<br>d. 41–50<br>e. 51–60<br>f. 61–70<br>g. 70+
	Q9. Do you currently experience any of the following medical conditions? (Please select all that apply)	a. Asthma<br>b. Lung condition other than asthma (chronic bronchitis, COPD or other)<br>c. Hay fever<br>d. Heart condition (heart failure, heart attack, angina)<br>e. Stroke or TIA<br>f. Diabetes<br>g. Pregnant<br>h. Other (please specify) {open text response}
	Q10. If you are a parent of a child/ren, what age is your child/ren? (Please select all that apply)	• 0–5 years old<br>• 5–10 years old<br>• 10–15 years old<br>• I am not a parent of a child in the three age brackets listed
	Q11. For Port Macquarie and ACT, were you pregnant during the smoky periods in your area between November 2019 and January 2020?	a. Yes<br>b. No<br>c. Not sure<br>d. I do not reside in Port Macquarie or ACT

	Q12. For Tasmania, were you pregnant and experienced smoky periods in your area between January and February 2019?	a. Yes<br>b. No<br>c. Not sure<br>d. I do not reside in Tasmania
	Q13. What is the postcode for the suburb where you reside?	{Open text response}
	Q14. What is your personal income bracket?	a. Nil income<br>b. $1–$10,399<br>c. $10,400–$15,599<br>d. $15,600–$20,799<br>e. $20,800–$31,199<br>f. $31,200–$41,599<br>g. $41,600–$51,199<br>h. $52,000–$64,999<br>i. $65,000–$77,999<br>j. $78,000–$103,999<br>k. $104,000 or more
Closing statement	Thank you for taking the time to complete the pre-interview/ focus group discussion questionnaire.<br><br>We look forward to hearing your thoughts on AirRater during an interview or focus group discussion soon.<br><br>If you have any questions, or need to confirm details for your participation in an interview or focus group, please contact the AirRater team at <air.rater@utas.edu.au> or call on 1800 332 102.	n/a

Table S2. Interview schedule for semi-structured interviews with AirRater users

<table>
<tr><td colspan="3">Introduction
Thank you for agreeing to be a participant in this research project. As I mentioned when we spoke/I wrote, I am exploring user experiences of AirRater, particularly when, how and why AirRater is used.
The purpose of this conversation is to gain insight into your personal use of AirRater. I will record our conversation so that I take minimal notes and can accurately capture your responses.
Your responses to my questions will be used to evaluate the application and may be included in internal and external publications reporting on this research. Do you have any questions before we start?</td></tr>
<tr><th>Primary question/prompt</th><th>Additional questions</th><th>Clarifying questions</th></tr>
<tr><td>1. Use of AirRater
Pease tell me about your use of AirRater.</td><td>When and why do you use AirRater?
How would you describe your overall experience with AirRater?
Is there anything that prompts you to use AirRater?
Do you report symptoms?</td><td rowspan="4">Can you please tell me about your experience?

Can you please expand on this?

Can you please explain?

Can you please give me an example?</td></tr>
<tr><td>2. Features of AirRater
Please tell me about your experience with AirRater's features.</td><td>Is there anything about using AirRater that you find irritating?
Are there additional features that you would like to see AirRater offer?</td></tr>
<tr><td>3. Comprehension of and trust in AirRater
Please tell me about your understanding of the information that AirRater provides.</td><td>Do you use the pollen/air quality/temperature information?
How would you describe your understanding of the information AirRater provides?
Do you ever question the information provided by AirRater?
Are there other sources of information you use for pollen, air quality and/or temperature?</td></tr>
<tr><td>4. Self-management and AirRater
Please tell me about whether you have used AirRater to support your health since you have downloaded the application.</td><td>Does AirRater provide any information that assists you in managing any symptoms?
Are there other tools or sources of information that assist you in managing any symptoms?</td></tr>
</table>

<table>
<tr><td>5. Behavior change and AirRater
Please tell me about what you do once you have looked at the information provided by AirRater.</td><td>Do you use any of the information provided by AirRater to help you make decisions about your day?
Are there any factors that impact your ability to make choices or decisions about your day?</td><td rowspan="2">Can you please describe how you use it?</td></tr>
<tr><td>6. Translational capacity of AirRater
Please tell me about any discussions you have had about AirRater with other people.</td><td>Do you discuss AirRater with family and/or friends?
Do you discuss AirRater with any health professionals, like a doctor or pharmacist?
Would you recommend AirRater to anyone?</td></tr>
<tr><td colspan="3">7. Is there anything that we have not discussed that you think might be relevant or of interest?</td></tr>
<tr><td colspan="3">Closing
Thank you again for taking the time to speak with me today. Your perspectives are important and valuable in helping me to understand more about when, how and why you use AirRater. A reminder that your responses may be used in documents reporting about AirRater.
If you have any questions or think of anything else that you would like to tell me after our conversation today, please feel free to contact the AirRater team.</td></tr>
</table>

Table S3. Characterisation of AirRater using the BCW framework (Michie et al. 2011)
The framework links the model of behavior (i.e. essential behavioral conditions) and policy categories (first column) with intervention functions (first row). The black ticks indicate the current intervention functions that AirRater employs. The green ticks indicate the current policy categories that AirRater informs; this occurs through the utilisation of population health data collected via the app by government health and environment agencies that are affiliated with the collaboration. The red ticks indicate the potential intervention functions and policy categories AirRater could pursue in future to enhance the effectiveness of the app. See Table S4 for relevant definitions.

**Model of Behavior: Sources**	**Education**	**Persuasion**	**Incentivisation**	**Coercion**	**Training**	**Restriction**	**Environmental Restructuring**	**Modelling**	**Enablement**
Physical capability (C-Ph)					✓				
Psychological capability (C-Ps)	✓				✓				
Reflective motivation (M-Re)	✓	✓	✓						
Automatic motivation (M-Au)									
Physical opportunity (O-Ph)							✓		✓
Social opportunity (O-So)							✓		✓
**Policy categories**									
Comms/ Marketing	✓								
Guidelines	✓	✓							
Fiscal			✓						
Regulation	✓	✓							✓
Legislation									
Planning							✓		✓
Service provision					✓				

Table S4. Definitions for model of behavior, intervention functions and policy categories for the Behavior Change Wheel (Michie et al. 2011)

**Model of Behavior (Essential Behavioral Conditions)**	
Physical capability (C-Ph)	Capacity to undertake activity, achieved through physical skill development which is the focus of training or potentially through enabling interventions such as medication, surgery or prostheses
Psychological capability (C-Ps)	Capacity to undertake activity, achieved through imparting knowledge or understanding, training emotional, cognitive and/or behavioral skills or through enabling interventions such as medication
Reflective motivation (M-Re)	Neurological processes that lead to activity, achieved through increasing knowledge or understanding, eliciting positive (or negative) feelings about behavioral target
Automatic motivation (M-Au)	Neurological processes that lead to activity, achieved through associative learning that elicit positive (or negative) feelings and impulses or counter-impulses relating to the behavioral target, imitative learning, habit formation or direct influences on automatic motivational processes (e.g. via medication)
Physical opportunity (O-Ph)	Physical factors beyond the individual that enable or challenge activity, achieved through environmental change
Social opportunity (O-So)	Social factors beyond the individual that enable or challenge activity, achieved through environmental change
**Intervention functions**	
Education	Increasing knowledge or understanding
Persuasion	Using communication to induce positive or negative feelings or stimulate action
Incentivisation	Creating expectation of reward
Coercion	Creating expectation of punishment/cost
Training	Imparting skills
Restriction	Using rules to reduce the opportunity to engage in the target behavior (or to increase the target behavior by reducing the opportunity to engage in competing behaviors)
Environmental restructuring	Changing the physical or social context
Modelling	Providing an example for individuals to aspire to or imitate
Enablement	Increasing means/reducing barriers to increase capability (beyond education and training) or opportunity (beyond environmental restructuring)
**Policy categories**	
Comms/marketing	Using print, electronic, telephonic or broadcast media
Guidelines	Creating documents that recommend or mandate practice. This includes all changes to service provision
Fiscal	Using the tax system to reduce or increase the financial cost

Regulation	Establishing rules or principles of behavior or practice
Legislation	Making or changing laws
Environmental/social planning	Designing and/or controlling the physical or social environment
Service provision	Delivering a service

Table S5. Summary of participant characteristics stratified by question

Characteristic	Respondents
**Downloaded AirRater (*n* = 38)**	
Less than 6 months ago	8 (21%)
6–12 months ago	13 (34%)
1–2 years ago	6 (16%)
2–3 years ago	5 (13%)
3–4 years ago	5 (13%)
4–5 years ago	1 (3%)
**Medical conditions (*n* = 32)**	
Asthma	17 (53%)
Lung condition (e.g. COPD)	9 (28%)
Allergic rhinitis	24 (75%)
Heart condition	3 (9%)
Stroke	0
Diabetes	2 (6%)
Pregnant	0
Other	12 (38%)
**Gender (*n* = 38)**	
Male	8 (21%)
Female	30 (79%)
Other	0
Prefer not to say	0
**Age range (*n* = 38)**	
18–20	0
21–30	3 (8%)
31–40	8 (21%)
41–50	4 (11%)
51–60	8 (21%)
61–70	9 (23%)
70+	6 (16%)
Prefer not to say	0
**Personal income bracket (*n* = 38)**	
Nil income	0
$1–$10,399	0
$10,400–$15,599	1 (3%)
$15,600–20,799	1 (3%)
20,800–$31,199	7 (18%)
$31,200–$41,599	2 (5%)
$41,600–$51,199	4 (11%)
$52,000–$64,999	3 (8%)
$65,000–$77,999	4 (11%)
$78,000–$103,999	7 (18%)
$104,000+	3 (8%)
Prefer not to say	6 (16%)
